# Diaphragmatic Paresis and Respiratory Failure After Interscalene Block in a Hypoxemic Trauma Patient: A Case Report

**DOI:** 10.7759/cureus.88387

**Published:** 2025-07-20

**Authors:** Ryosuke Fukuoka, Taku Mayahara, Tomohiro Katayama, Masao Uchihashi, Yuya Hirai

**Affiliations:** 1 Department of Anesthesiology, Kobe Ekisaikai Hospital, Kobe, JPN; 2 Department of Emergency and General Medicine, Kobe Ekisaikai Hospital, Kobe, JPN

**Keywords:** diaphragmatic paresis, hypoxemia, interscalene brachial plexus block, pneumothorax, regional anesthesia, respiratory failure, trauma

## Abstract

Interscalene brachial plexus block (ISB) is frequently employed for analgesia in clavicle surgery but can cause ipsilateral diaphragmatic paresis, potentially leading to respiratory compromise in patients with limited pulmonary reserve. Trauma patients with pneumothorax may undergo regional anesthesia with spontaneous ventilation to avoid positive pressure ventilation (PPV), but this approach carries significant risk if respiratory function is already compromised. A woman in her 60s with multiple rib fractures, a small pneumothorax, and persistent hypoxemia underwent ISB for clavicle surgery. To avoid PPV, general anesthesia with spontaneous ventilation was performed using a laryngeal mask airway. Soon after induction, oxygenation worsened, and postoperative chest imaging revealed right diaphragmatic elevation consistent with phrenic nerve involvement. Emergent intubation and PPV were required. Further investigation identified a previously undiagnosed pulmonary embolism as a contributing factor to persistent hypoxemia. This case highlights the need for careful preoperative evaluation of respiratory reserve and thromboembolic risk when considering ISB in hypoxemic trauma patients. Strategies aimed at avoiding PPV may inadvertently increase the risk of respiratory decompensation in patients with limited respiratory capacity.

## Introduction

Interscalene brachial plexus block (ISB) is commonly used for perioperative analgesia in clavicle surgery [1]. However, ISB is associated with a high incidence of ipsilateral diaphragmatic paresis [2], which can lead to respiratory compromise in patients with limited pulmonary reserve [3]. In trauma patients with pneumothorax, current guidelines recommend chest tube placement before general anesthesia with positive pressure ventilation (PPV) to reduce the risk of worsening pneumothorax or tension physiology [4]. Many patients with traumatic pneumothorax can be managed conservatively without chest tube placement. When surgery is required in such cases, strategies combining spontaneous ventilation with regional anesthesia techniques, such as ISB, may be chosen to avoid PPV [5]. We report a case of acute respiratory failure that developed in a trauma patient with persistent preoperative hypoxemia. The respiratory failure occurred following general anesthesia with spontaneous ventilation combined with ISB, highlighting critical considerations for perioperative risk assessment.

## Case presentation

**Figure 1:**
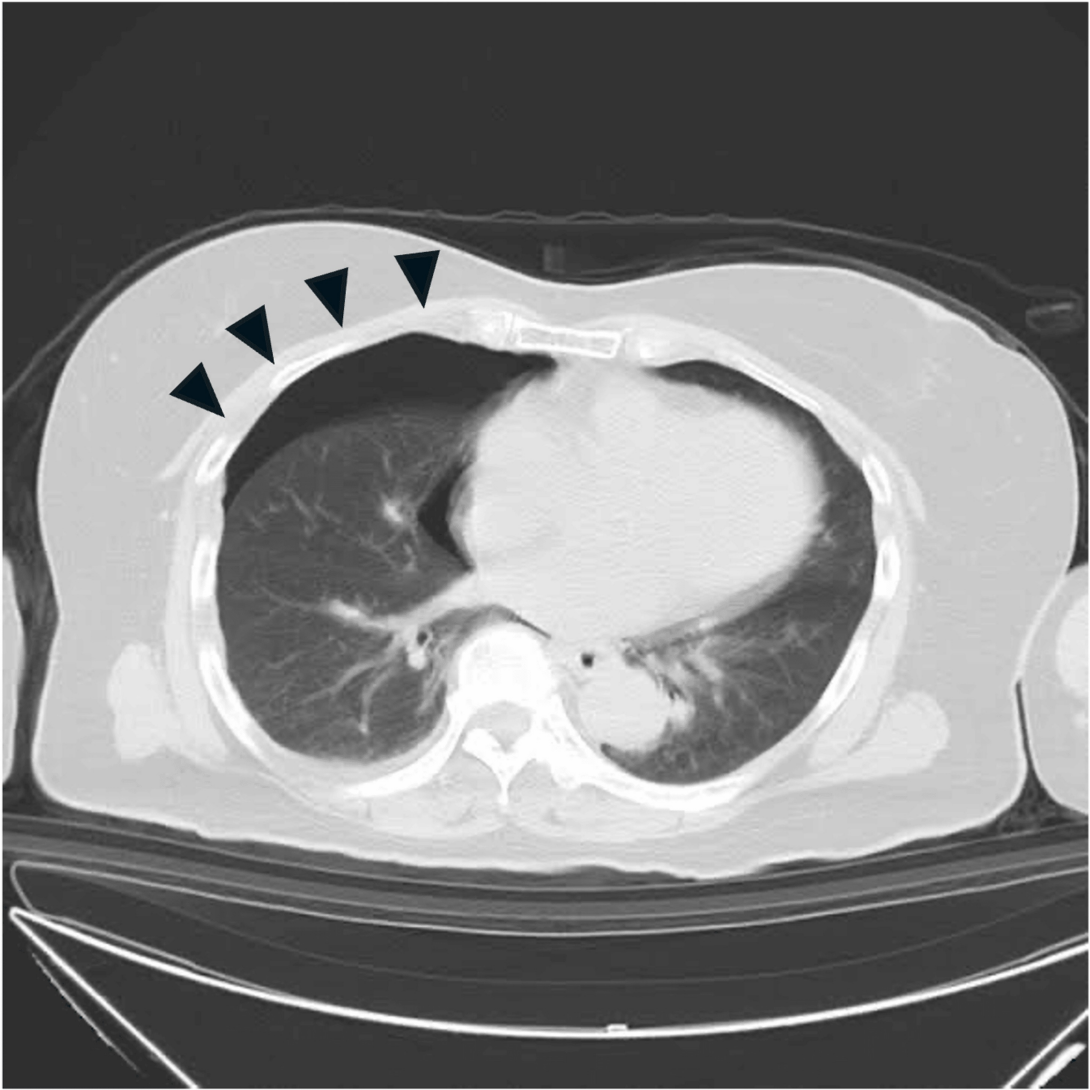
Initial chest computed tomography (CT). Initial chest CT revealed a right-sided pneumothorax (black arrowhead).

A woman in her 60s (height 155 cm, weight 65 kg) with a history of hypertension was brought to the emergency department following a motorcycle accident. On arrival, her vital signs were as follows: blood pressure 201/120 mmHg, heart rate 107 bpm, respiratory rate 20 breaths/minute, peripheral capillary oxygen saturation (SpO₂) 94%, and body temperature 36.8 °C. The patient exhibited agitation. Initial head computed tomography (CT) revealed a small intracerebral hemorrhage in the right parietal lobe. Chest imaging revealed fractures of the right clavicle and the second to fourth ribs, along with a right-sided pneumothorax (Figure 1) and a small hemothorax.

**Figure 2:**
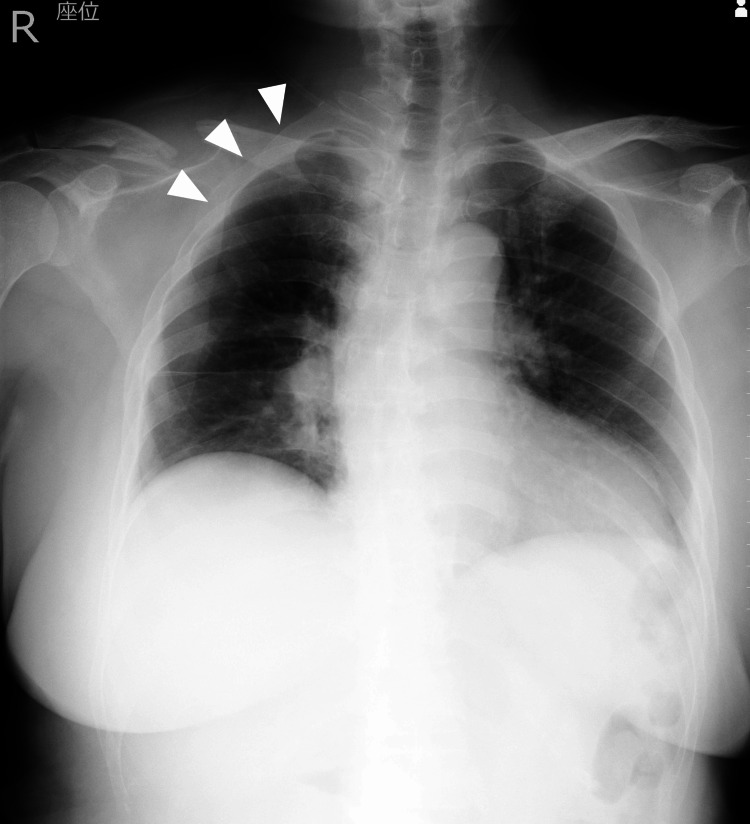
Preoperative chest radiograph (day before surgery). No recurrence of pneumothorax was observed, and there were no findings suggestive of pneumonia or atelectasis to explain the hypoxemia (white arrowhead).

No subcutaneous emphysema or pulmonary contusion was observed. A chest tube was inserted shortly after arrival. Subsequent imaging confirmed adequate re-expansion of the right lung and resolution of the air leak. After transfer to the intensive care unit, the patient remained agitated. The patient reported significant pain, which was presumed to contribute to the ongoing agitation. Initial analgesia with intravenous flurbiprofen and oral loxoprofen was inadequate; therefore, continuous fentanyl infusion at 25 μg/hour was initiated. Although analgesia improved, agitation persisted, requiring administration of haloperidol and dexmedetomidine. Oxygenation gradually deteriorated, and oxygen therapy via nasal cannula at 2 L/min was initiated. Fentanyl was discontinued on hospital day 2, and oral celecoxib was initiated for pain control. Risperidone was also introduced to manage agitation. Despite these interventions, nocturnal dexmedetomidine infusions were intermittently necessary. Follow-up head CT showed no progression of the intracerebral hemorrhage, and the patient's family reported no prior psychiatric history. The chest tube was removed on hospital day 5 following confirmation of lung re-expansion, primarily due to concerns about potential self-removal related to persistent agitation. Oxygenation continued to decline over the subsequent days. Chest radiography showed no evidence of recurrent pneumothorax, pneumonia, or atelectasis (Figure 2).

D-dimer levels were 5.3 μg/mL on day 1 and 1.7 μg/mL on day 3 (reference range: <1.0 μg/mL), with no upward trend. There were no signs of leg swelling indicative of deep vein thrombosis (DVT). At this stage, the cause of hypoxemia was considered multifactorial, including reduced thoracic mobility from rib fractures, mild obesity, and the effects of sedation. Although rehabilitation was initiated on hospital day 2, mobilization was limited due to patient refusal, sedation-related somnolence, and persistent hypoxemia. Despite the high risk of thrombosis, anticoagulant therapy was not administered due to concerns about worsening the cerebral hemorrhage.

**Figure 3:**
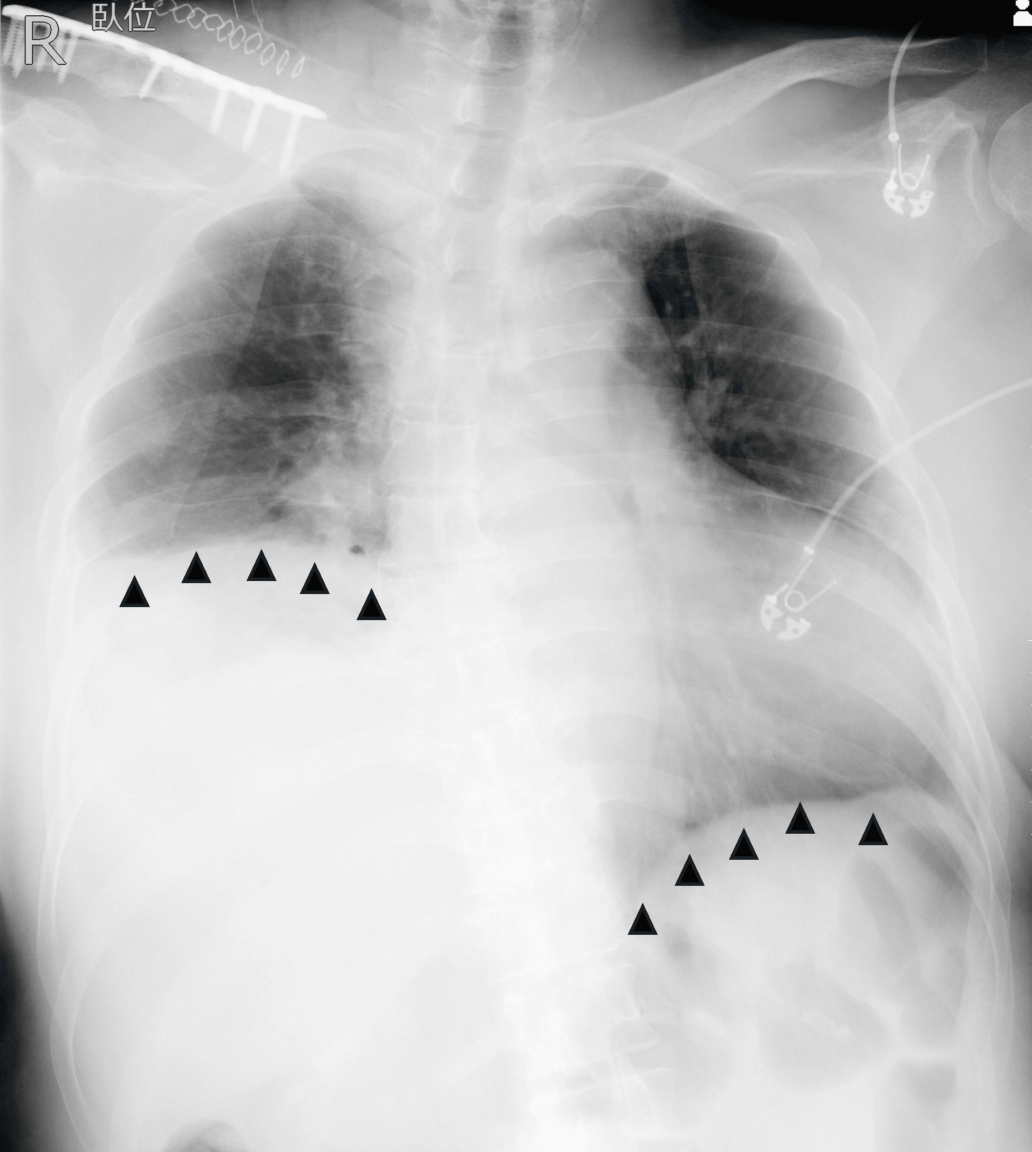
Postoperative chest radiograph. The right hemidiaphragm was significantly elevated compared to the left hemidiaphragm (black arrowhead).

Surgical fixation of the clavicle was scheduled for hospital day 9. By that time, the patient required 3-4 L/minute of oxygen via nasal cannula to maintain SpO₂ at 95%. General anesthesia was deemed necessary due to persistent agitation. To reduce the risk of barotrauma from positive pressure ventilation, a strategy involving spontaneous respiration with a supraglottic airway was selected. To facilitate intraoperative management, an ISB was also planned. Anesthesia was induced with 100 mg of propofol, and a laryngeal mask airway (LMA) was inserted. With the patient in the supine position, an ultrasound-guided ISB was performed via a posterior approach using 15 mL of 0.75% ropivacaine. Anesthesia was maintained with sevoflurane at 1.5% in 50% oxygen. Fentanyl was administered in 10 μg increments as needed (total dose: 100 μg) to maintain spontaneous ventilation at 10-20 breaths/minute. The patient underwent open reduction and internal fixation of the right clavicle in the beach-chair position. Shortly after the start of surgery, a gradual decrease in oxygen saturation was observed. SpO₂ could not be maintained above 95% at a fraction of inspired oxygen (FiO₂) of 0.5, and FiO₂ was increased to 1.0 to maintain SpO₂ between 97% and 100%. Attempts to reduce FiO₂ consistently resulted in decreased saturation; thus, 100% oxygen was maintained throughout most of the 120-minute procedure. Hemodynamics remained stable, and the procedure was completed without incident. Following emergence from anesthesia in the operating room, the LMA was removed. The patient subsequently developed marked agitation and hypoxemia, with SpO₂ around 90% despite oxygen administration at 10 L/min via face mask. Postoperative chest radiography demonstrated right-sided diaphragmatic elevation (Figure 3), suggesting diaphragmatic paresis as a contributing factor to her respiratory compromise.

**Figure 4:**
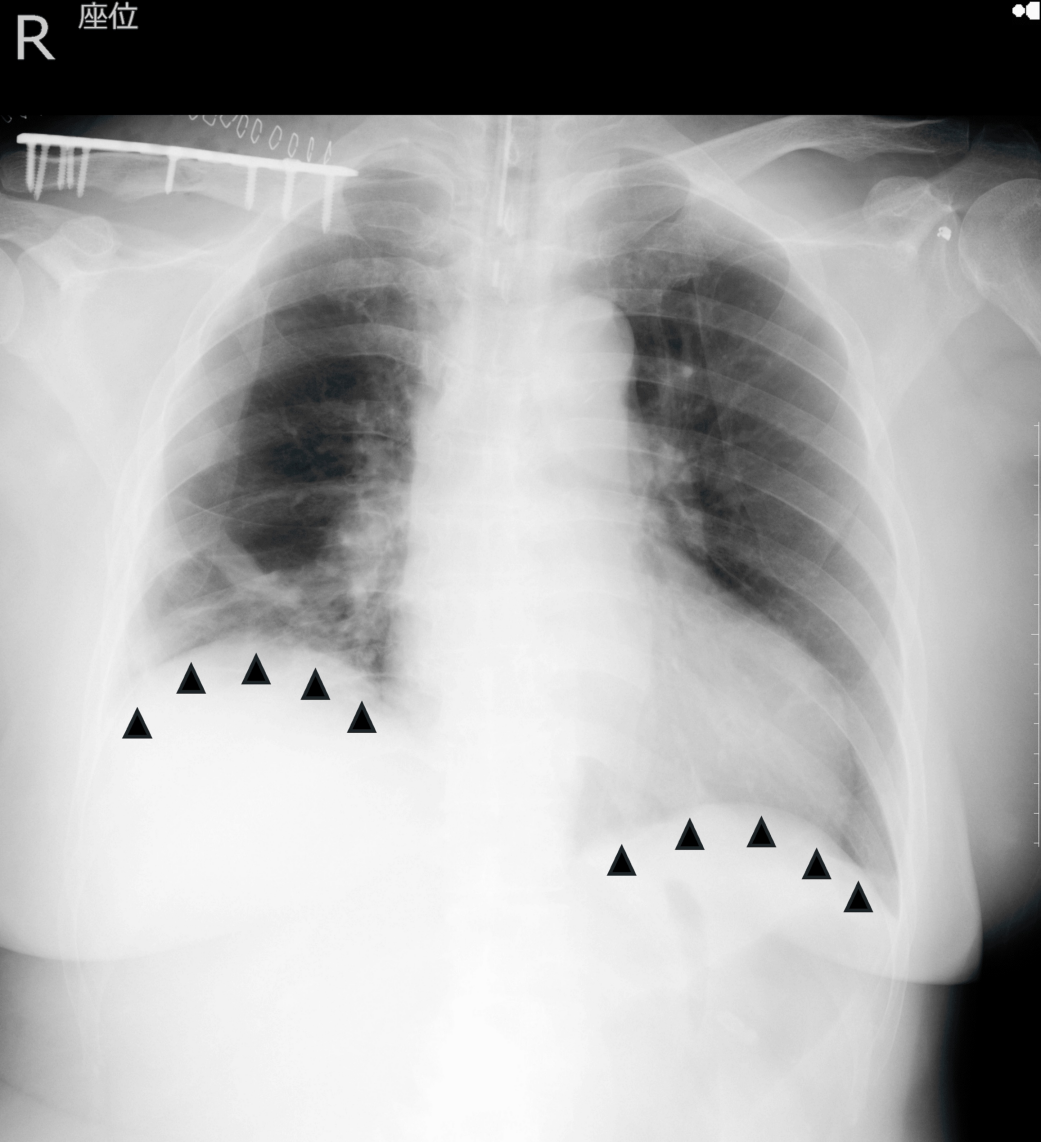
Chest radiograph after intubation and positive pressure ventilation. Improvement in right hemidiaphragmatic elevation was observed (black arrowhead).

Despite transitioning to a reservoir mask, oxygenation continued to deteriorate further. Arterial blood gas analysis revealed hypoxemia without hypercapnia. The arterial partial pressure of oxygen (PaO₂) was 48.6 mmHg (reference range: 75-100 mmHg), and the arterial partial pressure of carbon dioxide (PaCO₂) was 39.6 mmHg (reference range: 35-45 mmHg). Endotracheal intubation and PPV were initiated, resulting in improved oxygenation (PaO₂/FiO₂ >300). Follow-up imaging showed no recurrence of pneumothorax and improvement in right hemidiaphragmatic elevation (Figure 4).

The patient was extubated the following morning.

**Figure 5:**
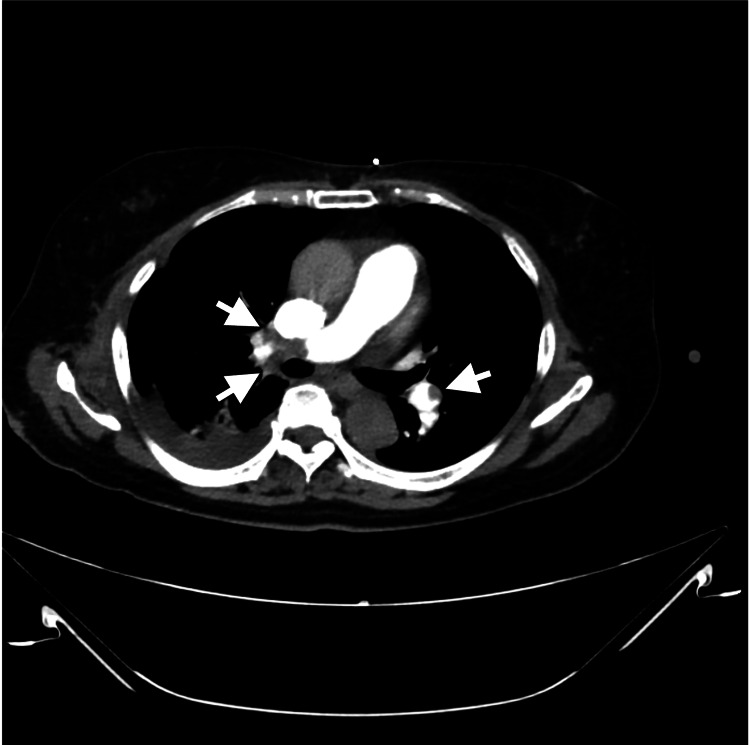
Contrast-enhanced chest CT. Filling defects were detected in the pulmonary arteries (white arrow), consistent with pulmonary embolism.

After extubation, high-flow nasal oxygen therapy (FiO₂ 30%-40%) remained necessary, and agitation persisted, requiring continued sedation. On hospital day 15, respiratory function deteriorated again, necessitating reintubation. Laboratory testing revealed a D-dimer level of 70.3 μg/mL. Contrast-enhanced CT identified pulmonary embolism (PE) (Figure 5) and DVT in the left lower extremity.

Anticoagulation therapy with heparin was initiated. Both respiratory function and neuropsychiatric status subsequently improved. The patient was extubated on hospital day 17, oxygen therapy was discontinued by day 21, and she was discharged home without sequelae on hospital day 48.

## Discussion

In this case, performing ISB and the resulting diaphragmatic paresis in a patient with persistent preoperative hypoxemia contributed to the development of acute respiratory failure. Several alternative strategies could have been considered to minimize the risk of diaphragmatic paresis. First, proceeding with general anesthesia under spontaneous ventilation without regional block and managing pain with systemic analgesia could be an option. Second, performing ISB with lower volumes of local anesthetic has been reported to reduce the incidence of diaphragmatic paresis [6], potentially allowing adequate analgesia while minimizing the risk of respiratory failure. Third, alternative regional techniques with less risk of diaphragmatic paresis, such as supraclavicular or superior trunk blocks, may be more suitable for patients with compromised respiratory reserve [7].

Current guidelines recommend chest tube placement before initiating PPV in trauma patients with pneumothorax to minimize the risk of worsening the pneumothorax or inducing tension physiology [4]. In the present case, the chest tube had already been removed before surgery, and the anesthetic plan focused on avoiding PPV by preserving spontaneous ventilation. However, given the persistent hypoxemia and respiratory instability, proceeding with general anesthesia under PPV after re-inserting a chest tube could be considered as an alternative strategy. Moreover, although controversial, some reports suggest that PPV without chest tube placement may be feasible in carefully selected cases of small, stable traumatic pneumothoraces [8,9]. Therefore, proceeding with PPV under general anesthesia without chest tube placement might also have been an alternative strategy in this case, with readiness to insert a chest tube intraoperatively if pneumothorax progressed.

Another important consideration was the delayed recognition of PE as a contributing factor to persistent hypoxemia. Although initial D-dimer levels were not elevated and there were no signs of DVT, the patient had multiple risk factors for venous thromboembolism, including trauma, immobility, and sedation. Early suspicion, timely prophylaxis, and appropriate diagnostic imaging should be considered when unexplained hypoxemia persists in trauma patients [10]. Additionally, persistent agitation significantly complicated both clinical assessment and management. Uncontrolled delirium can interfere with neurological and respiratory monitoring, delay mobilization, and hinder rehabilitation, all of which may contribute to suboptimal clinical decisions and outcomes. Effective delirium control is essential to optimize perioperative care for trauma patients with respiratory compromise [11]. Although head CT showed no new findings, brain magnetic resonance imaging was not performed to evaluate for possible central nervous system microembolism. Given the subsequent diagnosis of PE, the possibility of microemboli to the brain contributing to the patient’s altered mental status cannot be entirely excluded.

In summary, this case highlights the importance of carefully evaluating respiratory reserve and considering alternative regional techniques or strategies for pain management. It also highlights the importance of timely assessment and prophylaxis for thromboembolic events, as well as effective delirium control, including evaluation of the etiology of acute-onset delirium, in trauma patients with hypoxemia undergoing surgery.

## Conclusions

In trauma patients with preoperative hypoxemia, the use of ISB may result in diaphragmatic paresis, leading to acute respiratory failure. This case illustrates that strategies aimed at avoiding PPV can inadvertently increase the risk of respiratory compromise when regional techniques with known respiratory effects are used in patients with limited pulmonary reserve. A comprehensive evaluation of respiratory status and thromboembolic risk should be undertaken before selecting anesthetic techniques in this population.
